# Physiological Changes and Transcriptomics of *Elodea nuttallii* in Response to High-Temperature Stress

**DOI:** 10.3390/biology14080993

**Published:** 2025-08-04

**Authors:** Yanling Xu, Yuanyuan Jin, Manrong Zha, Yuhan Mao, Wenqiang Ren, Zirao Guo, Yufei Zhang, Beier Zhou, Tao Zhang, Qi He, Shibiao Liu, Bo Jiang

**Affiliations:** 1 College of Biology and Environmental Sciences, Jishou University, Jishou 416000, China; x1359152376@163.com (Y.X.); zmr0729@163.com (M.Z.); liushibiao_1@163.com (S.L.); 2 College of Biology and Food Engineering, Suzhou University of Technology, Suzhou 215500, China; 15751610676@163.com (Y.J.); m2757750955@163.com (Y.M.); wwlyl885522@163.com (Y.Z.); z2383890456@163.com (B.Z.); zhangtao@szut.edu.cn (T.Z.); 3 Fisheries Technology Extension Station of Changshu City, Suzhou 215500, China; kevinrenwq@163.com (W.R.); guozirao@126.com (Z.G.); 18762994260@163.com (Q.H.)

**Keywords:** *Elodea nuttallii*, high-temperature stress (HTS), physiological indicators, differentially expressed genes (DEGs), transcriptome, dormancy strategy

## Abstract

*Elodea nuttallii* is a significant submerged macrophyte utilized in shrimp and crab aquaculture, yet it exhibits low thermotolerance. This study investigated the physiological responses and transcriptomic characteristics of *E. nuttallii* under high-temperature stress (HTS). The results indicated that HTS significantly reduced the absolute growth rate (AGR) and photosynthetic efficiency of *E. nuttallii* while concurrently elevating antioxidant enzyme activities, malondialdehyde (MDA) content, and concentrations of osmotic adjustment compounds. Furthermore, the apical segments of *E. nuttallii* demonstrated greater sensitivity to HTS compared to the middle segments. Under exposure to 35 °C and 40 °C, antioxidant enzyme activities, MDA content, and osmotic adjustment compound levels were significantly higher in the apical segments than in the middle segments. Transcriptomic analysis revealed 7526 differentially expressed genes (DEGs) in the apical segments at 35 °C, a number substantially exceeding that observed in the middle segments. Enrichment analysis of DEGs revealed significant upregulation of key metabolic regulators under HTS, including carbohydrate metabolism genes (*HXK*, *FRK*) and phenylpropanoid biosynthesis enzymes (*4CL*, *COMT*). This transcriptional reprogramming demonstrates *E. nuttallii*’s adaptive strategy of modulating carbon allocation and phenolic compound synthesis to mitigate thermal damage. Our findings not only elucidate novel thermotolerance mechanisms in aquatic plants but also provide candidate genetic targets (*HXK*, *4CL*) for molecular breeding of heat-resilient cultivars through transcriptomic screening.

## Simple Summary

Yangcheng Lake’s famous Chinese mitten crab industry faces a severe threat: summer heatwaves destroy the underwater plants crucial for crab habitat. These plants, called *Elodea nuttallii*, provide essential shelter for crabs but collapse during heatwaves when their stems break at the base, causing large patches to detach and die off. Since crabs depend completely on these plants, this leads to massive crab deaths and reduced harvests. To address this crisis, we investigated how these plants survive high temperatures. By studying their reactions to heat stress, we discovered that they employ a special survival strategy: swiftly activating self-defense systems to prevent heat damage while simultaneously reducing their energy use to enter an “energy-saving mode”. These insights reveal the plants’ hidden resilience tactics against extreme heat and provide the scientific foundation for breeding heat-resistant varieties. Ultimately, this research will help local crab farmers protect aquatic habitats against climate warming, secure sustainable crab production at Yangcheng Lake, and safeguard the future of this treasured Chinese food tradition for generations.

## 1. Introduction

The Chinese mitten crab (*Eriocheir sinensis* H. Milne-Edwards, CMC) is a hairy crab that is produced in the Yangcheng Lake area of Suzhou, China. It is the most popular and valuable type of hairy crab and has been recognized by the Ministry of Agriculture and Rural Affairs of China as a landmark agricultural product brand in China [1]. *Elodea nuttallii* (Planch.) H. St. John plays a very important role in the artificial breeding of CMC [2].

*Elodea nuttallii* is a submerged plant that is native to the Americas and belongs to the family Hydrocharitaceae [3]. Because of its rapid growth, fast reproduction, and high yield, it has been widely used in CMC aquaculture since the 1990s [4]. *E. nuttallii* provides a hiding place for crabs during shelling, which is a period of rapid growth in crab weight [5]. In addition, *E. nuttallii* can absorb excess nutrients in the water to repair the water body for cultivation, and a large amount of *E. nuttallii* can help maintain a lower temperature in the water body [6]. These characteristics can help crabs accelerate their growth, reduce the occurrence of diseases, aid crabs in safely surviving the hot season, and improve the yield and quality of crabs [7]. As a cryophilic macrophyte, *E. nuttallii* is indigenous to North American freshwater habitats characterized by high irradiance yet low thermal regimes. Its optimal growth temperature ranges from 10 °C to 25 °C, with pronounced thermosensitivity beyond this threshold [8,9]. In summer, the air and water temperatures in Yangcheng Lake usually reach 35–40 °C and 26–34 °C [10], respectively, and the growth of a large number of *E. nuttallii* becomes stagnant, with broken roots of *E. nuttallii* floating on the water surface under these high temperatures. In addition, *E. nuttallii* decays under continuous high temperatures, which leads to a serious deterioration of the water quality [11]. Summer coincides with the shelling period of crabs, and the reduction in shelter and the deterioration of water quality in their shelling hiding areas seriously affect the growth of hairy crabs in Yangcheng Lake, as well as their quality [12]. However, there have been no studies elucidating the stress mechanism of *E. nuttallii* in response to high-temperature stress (HTS).

HTS is one of the major environmental stresses that limit plants’ growth, metabolism, and productivity [13,14]. It occurs when the temperature exceeds the threshold level of the plant for a period of time and when the plant is not adequately regulated to resist the damage caused by HTS [15]. The in vivo accumulation of osmotic regulators is one of the main physiological mechanisms of plants in response to HTS [16]. Plants use proline (Pro), soluble sugar (SS), and soluble protein (SP) to maintain the moisture of cells and tissues, reduce the osmotic potential, and protect the integrity of cell membranes [17,18]. HTS accelerates the production of reactive oxygen species (ROS), and a large accumulation of ROS can cause damage such as DNA damage and lipid peroxidation [19]. Plants remove excess ROS from the body through two pathways, the antioxidant enzyme system and the non-enzymatic antioxidant system, while the enzyme reaction system can inhibit intracellular superoxide radicals (O_2_•^−^) and maintain their metabolic stability [20]. For most plants, as long as the temperature does not cause extensive damage to organelle membranes or necrotizing damage to leaves [21], the effect of high temperatures is reversible [22]. The degree and duration of recovery depend on the length and severity of the high-temperature treatment [23]. For example, the net CO_2_ uptake of ivy leaves was significantly reduced after treatment at 44 °C for 1–2 h and completely recovered after 7 days of treatment at 20 °C [24]. However, whether this reversible mechanism exists in *E. nuttallii* has not been reported.

RNA sequencing (RNA-seq) has been widely used to study plants’ responses to abiotic stresses [25]. Mangelsen et al. uncovered the impact of temperature on storage substance biosynthesis and regulation through a transcriptomic analysis of *Hordeum vulgare* L. under HTS [26]. *E. nuttallii*, a significant submerged macrophyte utilized in Chinese mitten crab (*Eriocheir sinensis*) aquaculture, has consequently garnered considerable research interest regarding its survival capacity under high-temperature stress. This study investigated alterations in physiological indices of *E. nuttallii* under HTS and integrated transcriptomic data to propose its underlying response mechanisms.

## 2. Materials and Methods

### 2.1. Plant Material and Growing Conditions

The *E. nuttallii* test plant was collected from a crab pond in Changshu City, Jiangsu Province, China. The *E. nuttallii* plant was washed with dechlorinated tap water to remove impurities, and the attached algae were separated with forceps. The clean plants were then cultured in buckets under laboratory conditions. Healthy *E. nuttallii* plants of about 15 cm in length were grown in buckets containing well-washed river sand and acclimatized under control conditions for one week. All experimental *E. nuttallii* specimens across the different temperature treatments were cultivated in standardized polyethylene containers (30 cm × 35 cm × 31.5 cm) containing 5% Hoagland nutrient solution (HNS, pH 6.8 ± 0.2), with the substrate consisting of sterilized river sand (particle size ≤ 2 mm through mechanical sieving). Each treatment group maintained triplicate biological replicates under controlled photoperiod conditions.

The *E. nuttallii* domestication incubator was set to 25 °C (room temperature as a control), 30 °C, 35 °C, and 40 °C. The culture environment was created using a light incubator (FQ-280B NJ, Feiqi, Nanjing, China). The treatment photoperiod was 12 h dark/12 h light, and the light intensity was 270–300 μmol/m^2^·s^1^ [27,28]. The samples were collected at 0 h, 24 h, 48 h, and 72 h and restored to 25 °C for 72 h. The samples were rapidly collected from the apical and middle portions and immediately frozen in liquid nitrogen. The samples were used for physiological and transcriptomic assays.

### 2.2. Growth Measurements

The growth parameters of *E. nuttallii* were measured and recorded at 0 h, 24 h, 48 h, and 72 h and after recovery to 25 °C for 72 h of cultivation under different temperature conditions, with three replicates being used per treatment. The initial and final lengths from the root to the apex of the plant were measured. The absolute growth rate (AGR) was calculated as follows: AGR=(FL−IL)/Time. Here, AGR denotes the absolute growth rate (units: cm·d^−1^), FL is the final length, IL is the initial length (units: cm), and Time indicates the growing time (units: d).

### 2.3. Antioxidant Enzyme Activity Assay

Superoxide dismutase (SOD) activity was quantified spectrophotometrically using nitroblue tetrazolium (NBT) reduction methodology with a commercial assay kit (kit series no. SOD-2-W, Suzhou Comin Biotechnology Co., Ltd., Suzhou, China) [29]. The superoxide anion (O_2_•^−^) reduces nitroblue tetrazole to generate blue formazan, for which the maximum absorption peak is 560 nm. SOD scavenges O_2_•^−^, which results in inhibition of formazan formation. The bluer the reaction liquid, the lower the SOD activity. Catalase (CAT) activity was assayed using a CAT assay kit (kit series no. CAT-2-W, Suzhou Comin Biotechnology Co., Ltd., Suzhou, China) employing the ammonium molybdate colorimetric method [30]. H_2_O_2_ has a characteristic absorption peak at 240 nm, and CAT can decompose H_2_O_2_, so the absorbance of the reaction solution at 240 nm decreased with the reaction time. CAT activity could then be calculated according to the rate of change in absorbance. Ascorbate peroxidase (APX) activity was determined using an APX reagent kit (kit series no. APX-2-W, Suzhou Comin Biotechnology Co., Ltd., Suzhou, China) based on APX catalyzing the reaction of ASA and H_2_O_2_ to oxidize ascorbic acid (ASA) [31]. APX activity was assayed spectrophotometrically by checking the oxidation rate of ASA at 25 °C. Peroxidase (POD) activity was determined using the guaiacol method with an assay kit (kit series no. POD-2-Y, Suzhou Comin Biotechnology Co., Ltd., Suzhou, China) [32]. POD catalyzes the oxidation of specific substrates with H_2_O_2_ and has a characteristic light absorption at 470 nm.

### 2.4. Determination of Malondialdehyde (MDA), Pro, SP, and SS Contents

Malondialdehyde (MDA) content was determined using the thiobarbituric acid (TBA) method (kit series no. MDA-2-Y, Suzhou Comin Biotechnology Co., Ltd., Suzhou, China) [30]. MDA combined with TBA to produce a red product with a maximum absorption peak at 532 nm. The content of lipid peroxide in the sample could be estimated after colorimetry; the MDA content was calculated as the difference between the absorbance values at 532 and 600 nm. Proline (Pro) content was assayed using a Pro assay kit (kit series no. Pro-2-Y, Suzhou Comin Biotechnology Co., Ltd., Suzhou, China) employing the sulfosalicylic acid (SSA) method [30]. Pro was extracted with sulfosalicylic acid and reacted with an acidic ninhydrin solution to produce a red color after heating. The absorbance value was measured at 520 nm after extraction with methylbenzene. SP content was measured using an SP assay kit (kit series no. BCAP-2-W, Suzhou Comin Biotechnology Co., Ltd., Suzhou, China) employing the bicinchoninic acid (BCA) method [33]. Under alkaline conditions, cysteine, tryptophan, tyrosine, and peptide bonds in proteins can reduce Cu^2+^ to Cu^+^. Two molecules of BCA combined with Cu^+^ to form a purple complex, which had an absorption peak at 562 nm. The absorbance values of a blank tube, standard tube, and measuring tube at 562 nm were recorded as Ab562 for the blank tube, As562 for the standard tube, and Am562 for the measuring tube. SS content was quantified using the anthrone method with an SS assay kit (kit series no. KT-2-Y, Suzhou Comin Biotechnology Co., Ltd., Suzhou, China) [33].

### 2.5. Chlorophyll Content Measurements

The chlorophyll content was determined using the spectrophotometric method described by Sumanta et al. [34]. Chlorophyll fluorescence values were measured using a portable chlorophyll fluorometer (MINI-PAM, Walz, Effeltrich, Germany). Fresh algal bodies were dried and darkened for 20 min, and their Fv/Fm values were determined by placing the bodies in a clamp.

### 2.6. RNA Sequencing

*E. nuttallii* treated at 35 °C for 24 h was collected for transcriptome sequencing (RNA-seq). To ensure the quality of the sequencing data, 18 samples (apical segments under 25 °C control conditions (T_CK), apical segments subjected to 35 °C thermostress exposure for 24 h (T_HS), apical segments undergoing post-thermostress recovery at 25 °C for 30 min (T_R), middle segments under 25 °C control conditions (M_CK), middle segments subjected to 35 °C thermostress exposure for 24 h (M_HS), and middle segments undergoing post-thermostress recovery at 25 °C for 30 min (M_R), each containing three biological repetitive sequences) were selected, frozen in liquid nitrogen, and quickly stored in a refrigerator at −80 °C for further RNA sequencing. The total RNA extracted from the leaves was analyzed for transcriptome analysis using TRIzol reagent (ThermoFisher, 15596018, Shanghai, China). The amount and purity of total RNA were measured using a NanoDrop ND-1000 (NanoDrop, Wilmington, DE, USA), and the integrity of the RNA was detected using a Bioanalyzer 2100 (Agilent, Santa Clara, CA, USA); concentrations > 50 ng/μL, RIN values > 7.0, and total RNA > 1 μg indicated successful downstream experiments. The sequencing tool used was the Illumina NovaSeq TM6000 (LC Bio Technology Co., Ltd., Hangzhou, China), which was used for bipartite sequencing according to standard procedures; the sequencing mode was PE150.

### 2.7. Statistical Analysis

Each experiment was performed in three independent biological and technical replicates. IBM SPSS Statistics 25.0 statistical software was used to analyze the pattern of change in the physiological parameters. One-way ANOVA (*p* < 0.05) was performed using Duncan’s test. SPSS statistical software was used for data entry and analysis. The mapping was drawn using Origin 2022 software, and the transcriptome correlation map was drawn through the cloud platform of Lianchuan Biotech (Hangzhou, China).

## 3. Results

### 3.1. Effects of High-Temperature Stress on Growth Parameters of E. nuttallii

**Figure 1:**
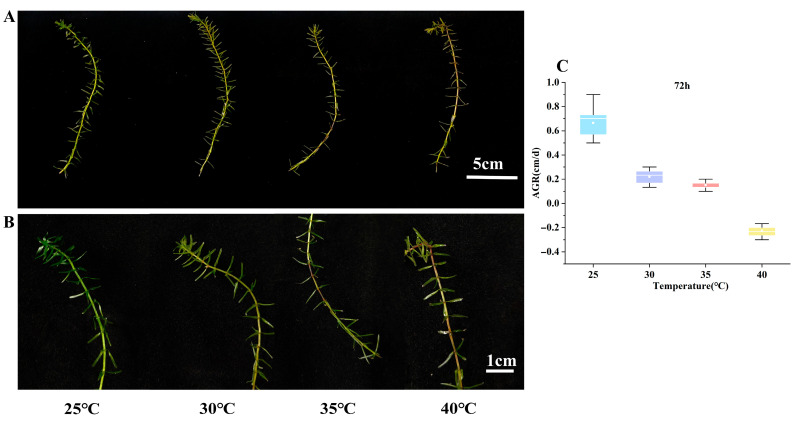
(**A**) Morphological changes in *E. nuttallii* after 72 h of growth under temperature stress: 25 °C—control group (CK); 30 °C—plants treated at 30 °C for 72 h; 35 °C—plants treated at 35 °C for 72 h; 40 °C—plants treated at 40 °C for 72 h. (**B**) Microstructural features of *E. nuttallii* after 72 h of growth under temperature stress. (**C**) Growth rate changes of *E. nuttallii* at different temperatures after 72 h. AGR: absolute growth rate.

Following 72 h of HTS, *E. nuttallii* exhibited no discernible localized lesions at 30 °C (Figure 1A,B). At 35 °C, localized lesions appeared, while structural integrity remained uncompromised (Figure 1B). Under 40 °C treatment, apical fracture occurred, with extensive lesions throughout the axial organs (Figure 1B). The AGR of *E. nuttallii* after 72 h of growth under temperature stress was significantly lower than that of the control group (Figure 1C, Table 1). Specimens subjected to 30 °C and 35 °C treatments exhibited attenuated axial elongation, whereas at 40 °C, sustained positive growth failed due to thermal damage, manifesting as tissue necrosis. Consequently, AGR values entered the negative-growth phase (Figure 1C, Table 1).

### 3.2. Changes in Antioxidant Enzyme Activities of E. nuttallii

**Figure 2:**
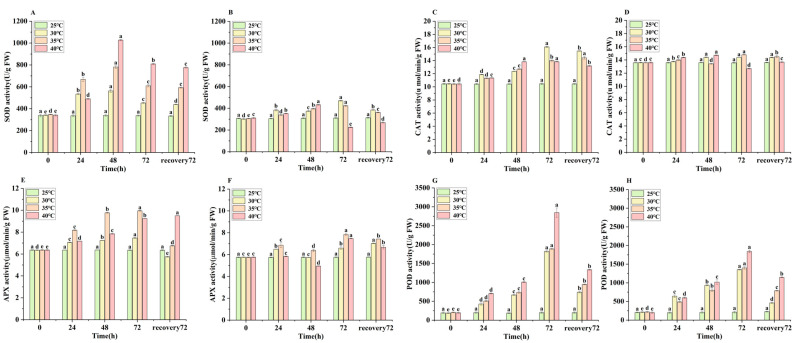
Changes in superoxide dismutase (SOD), catalase (CAT), ascorbate peroxidase (APX), and peroxidase (POD) activities in apical and middle segments of *E. nuttallii* under different temperature stress regimes: (**A**) apical SOD, (**B**) middle SOD, (**C**) apical CAT, (**D**) middle CAT, (**E**) apical APX, (**F**) middle APX, (**G**) apical POD, (**H**) middle POD. Values represent means ± SD (*n* = 3). Lowercase letters indicate statistically significant differences (*p* < 0.05). Time points: 0 h (pre-stress), 24 h, 48 h, and 72 h during temperature stress; 72 h recovery denotes 72 h at 25 °C following stress treatment. Temperature treatments: 25 °C, 30 °C, 35 °C, and 40 °C.

Under different temperature stress regimes, the activities of SOD, CAT, APX, and POD were significantly higher in the apical segments than in the middle segments (Figure 2). SOD activity in the apical segments of *E. nuttallii* exhibited similar trends across the three temperature treatments, with the most pronounced increase observed in the 40 °C treatment group (Figure 2A). In the middle segments, SOD activity increased significantly with prolonged exposure time under the 35 °C and 40 °C treatments. However, a significant decrease occurred after 72 h recovery at 25 °C following 35 °C stress (Figure 2B). CAT activity in the apical segments under 30 °C stress increased significantly over 72 h, reaching 16.08 U/g—representing a 53.73% increase over pre-stress levels. In contrast, apical CAT activity reached 13.98 U/g and 13.83 U/g at 72 h under the 35 °C and 40 °C treatments, respectively (Figure 2C).

APX activity in the apical segments increased non-significantly under 30 °C stress, reaching 7.47 μmol/min/g at 72 h (17.42% higher than pre-stress levels). Under 35 °C stress, apical APX activity reached 9.95 μmol/min/g at 72 h (56.31% higher than pre-stress levels). At 40 °C, apical APX activity increased significantly within 72 h and remained elevated after 72 h recovery at 25 °C (Figure 2E).

POD activity in both the apical and middle segments of *E. nuttallii* increased following 72 h of temperature stress. Apical POD activity increased by 841.39% (30 °C), 875.88% (35 °C), and 1372.44% (40 °C) compared to pre-stress levels. This activity decreased rapidly upon stress removal (Figure 2G). At peak activity (72 h), the apical POD levels exceeded those in the middle segments, showing increases of 554.83% (30 °C), 577.41% (35 °C), and 790.31% (40 °C) relative to the middle segment baselines (Figure 2H).

### 3.3. Changes in Malondialdehyde (MDA) Content of E. nuttallii

**Figure 3:**
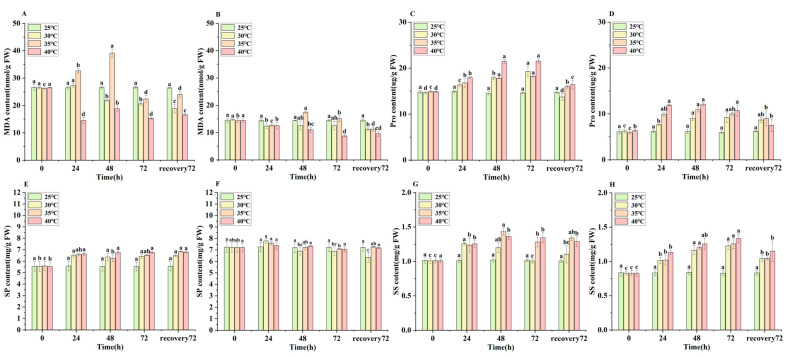
Changes in MDA, Pro, SP, and SS contents in different segments of *E. nuttallii* under temperature treatments: (**A**) apical MDA, (**B**) middle MDA, (**C**) apical Pro, (**D**) middle Pro, (**E**) apical SP, (**F**) middle SP, (**G**) apical SS, (**H**) middle SS. Values represent means ± SD (*n* = 3). Lowercase letters indicate statistically significant differences (*p* < 0.05). Time points: 0 h (pre-stress), 24 h, 48 h, and 72 h during temperature stress; 72 h recovery denotes 72 h at 25 °C following stress treatment. Temperature treatments: 25 °C, 30 °C, 35 °C, and 40 °C.

The MDA content in both the apical and middle segments of *E. nuttallii* exhibited an initial increase followed by a decrease across all temperature treatments (Figure 3). In the apical segments, the MDA content peaked at 48 h under both 30 °C and 35 °C stress (Figure 3A). The middle segments showed significant stress-induced variation: the MDA content increased substantially within 48 h under the 30 °C and 35 °C treatments, whereas a significant reduction occurred under 40 °C stress (Figure 3B).

### 3.4. Changes in the Contents of Osmoregulatory Compounds in E. nuttallii

In the apical segments, the Pro content exhibited an increasing trend under all temperature treatments. At peak accumulation (72 h), the Pro levels increased by 30.4% (30 °C), 22.3% (35 °C), and 45.3% (40 °C) relative to the pre-stress baselines. These elevated levels decreased following 72 h of recovery at 25 °C (Figure 3C). The middle segments contained a lower Pro content than the apical segments, exhibiting unimodal patterns that peaked at 48 h. At this peak, the Pro concentrations reached 9.0 μg/g (30 °C), 10.9 μg/g (35 °C), and 12.1 μg/g (40 °C), representing increases of 48.2%, 78.7%, and 98.4%, respectively, over the pre-stress values. A subsequent progressive decline occurred after 48 h (Figure 3D).

Under temperature stress, the SP content in the apical segments of *E. nuttallii* increased significantly within 72 h. In the 30 °C, 35 °C, and 40 °C treatment groups, the apical SP content reached 6.42 mg/g, 6.53 mg/g, and 6.77 mg/g, respectively, representing increases of 16.09%, 16.82%, and 22.64% compared with the pre-stress levels. The apical SP content did not change significantly after the removal of temperature stress (Figure 3E). The SP content in the middle segments was higher than that in the apical segments. After peaking at 24 h, the middle-segment SP content decreased. Compared with the pre-stress levels, the SP content in the middle segments of the 30 °C, 35 °C, and 40 °C treatment groups increased by 7.76%, 5.12%, and 2.5%, respectively (Figure 3F).

The SS content in the apical segments of the 30 °C treatment group initially increased, reached a peak at 24 h, and subsequently decreased significantly. In contrast, the SS content in the 35 °C and 40 °C treatment groups increased significantly within 48 h, reaching levels that were 18.95% and 34.65% higher than the pre-stress levels, respectively (Figure 3G). The SS content in the middle segments increased significantly within 72 h across all three temperature treatment groups but decreased markedly after 72 h upon resumption of the 25 °C treatment (Figure 3H).

### 3.5. Changes in Chlorophyll Contents and Chlorophyll Fluorescence of E. nuttallii

**Figure 4:**
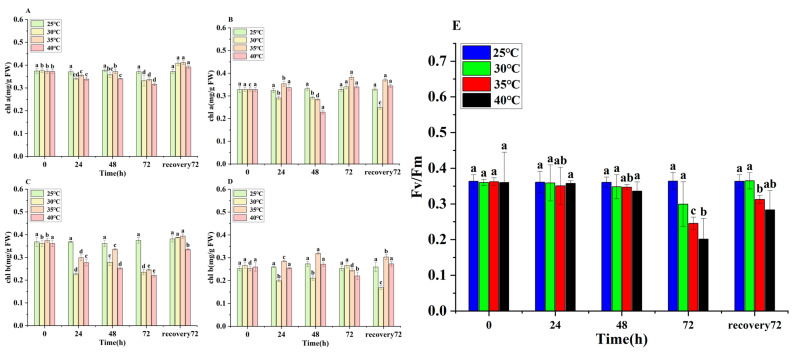
Effects of temperature stress treatments on chlorophyll contents in apical and middle segments of *E. nuttallii*: (**A**) apical chl a, (**B**) middle chl a, (**C**) apical chl b, (**D**) middle chl b, (**E**) Fv/Fm. Values represent means ± SD (*n* = 3). Lowercase letters indicate statistically significant differences (*p* < 0.05). Time points: 0 h (pre-stress), 24 h, 48 h, and 72 h during temperature stress; 72 h recovery denotes 72 h at 25 °C following stress treatment. Temperature treatments: 25 °C, 30 °C, 35 °C, and 40 °C.

The chlorophyll a (chl a) and chlorophyll b (chl b) contents in *E. nuttallii* decreased significantly under temperature stress. At 72 h, the chl a content in the apical segments in the 30 °C, 35 °C, and 40 °C treatment groups was 0.33 mg/g, 0.33 mg/g, and 0.31 mg/g, respectively, representing reductions of 10.81%, 10.81%, and 16.21% from the pre-stress levels (Figure 4A). Similarly, the chl b content in the apical segments reached 0.23 mg/g, 0.23 mg/g, and 0.22 mg/g at 72 h, corresponding to decreases of 36.11%, 35.14%, and 38.89% relative to the pre-stress values (Figure 4C). Chlorophyll fluorescence analysis revealed a decline in Fv/Fm values under temperature stress. In the 40 °C treatment group at 72 h, Fv/Fm decreased to 0.2, suggesting significant inhibition of photosynthesis by HTS (Figure 4E).

### 3.6. Transcriptome Analysis of Gene Expression in E. nuttallii Under HTS

Phenotypic analysis conducted under HTS determined that 40 °C represents the lethal temperature for *E. nuttallii*. Since 35 °C approximates summer temperatures in aquaculture crab ponds, this temperature was selected for RNA sequencing to investigate the temperature stress response mechanisms in *E. nuttallii*. Detailed quality metrics, including reference genome alignment rates, functional annotation outcomes, and Q30 scores, are comprehensively documented in Tables S1–S5 in the Supplementary Materials. These results confirm that the base-calling accuracy thresholds were rigorously met, validating suitability for subsequent bioinformatic analyses.

**Figure 5:**
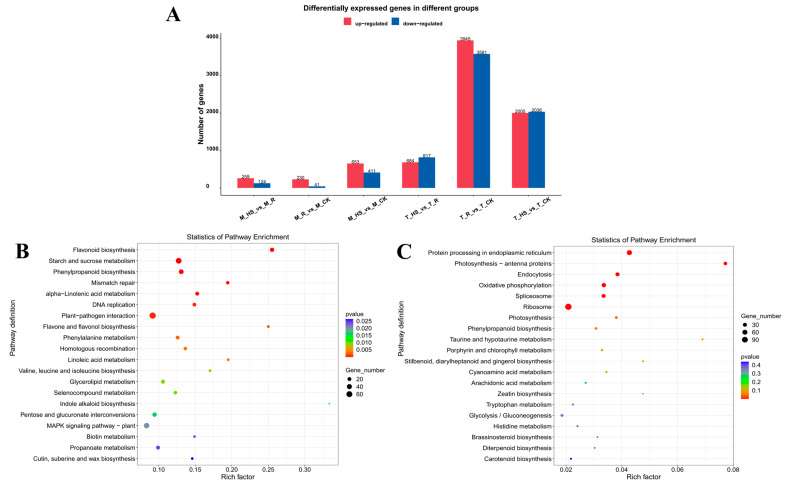
Analysis of differentially expressed genes. (**A**) Identification of differentially expressed genes. (**B**) The KEGG enrichment analysis between T_CK and T_HS. (**C**) The KEGG enrichment analysis between M_CK and M_HS.

The DEGs of the apical and middle segments of *E. nuttallii* were counted after the application of temperature stress and the recovery control treatment (treatment at 35 °C for 24 h and recovery at 25 °C for 30 min), and the DEGs of the two parts of *E. nuttallii* were taken as the control for 0 h. The changes in the DEGs in the two parts of *E. nuttallii* in the two stages of the temperature stress treatment are shown in Figure 5. Based on the following thresholds (DEGs, *p* < 0.05, |log2(FC)| ≥ 1): 2005 up-DEGs and 2036 down-DEGs between T_HS and T_CK; 3945 up-DEGs and 3581 down-DEGs between T_R and T_CK; 684 up-DEGs and 817 down-DEGs between T_HS and T_R; 653 up-DEGs and 411 down-DEGs between M_CK and M_HS; 230 up-DEGs and 41 down-DEGs between M_R and M_CK; and 259 up-DEGs and 124 down-DEGs between M_HS and M_R (Figure 5A).

In order to further explore the function of these DEGs, KEGG enrichment analysis was performed on the significantly regulated DEGs in each treatment group. KEGG pathway enrichment analysis showed that 443 of the 4041 DEGs in the apical segments of *E. nuttallii* were significantly enriched in 20 KEGG pathways (*p* < 0.05) under HTS, while only 312 of the 1064 DEGs in the middle segments of *E. nuttallii* were significantly enriched in 7 KEGG pathways (*p* < 0.05) under HTS. Among the 27 apical-segment KEGG-enriched pathways, flavonoid biosynthesis, phenylpropanoid biosynthesis, cutin, suberin and wax biosynthesis, protein processing in the endoplasmic reticulum, and photosynthesis-antenna proteins were common pathways in the apical and middle segments under HTS (Figure 5B,C). In addition, DEGs from the apical segments of *E. nuttallii* were significantly enriched in carbohydrate metabolic pathways such as starch and sucrose metabolism, pentose and glucuronate interconversions, and glycolysis/gluconeogenesis under HTS (Figure 5B,C).

### 3.7. Analysis of Differentially Expressed Genes Related to Photosynthesis

**Figure 6:**
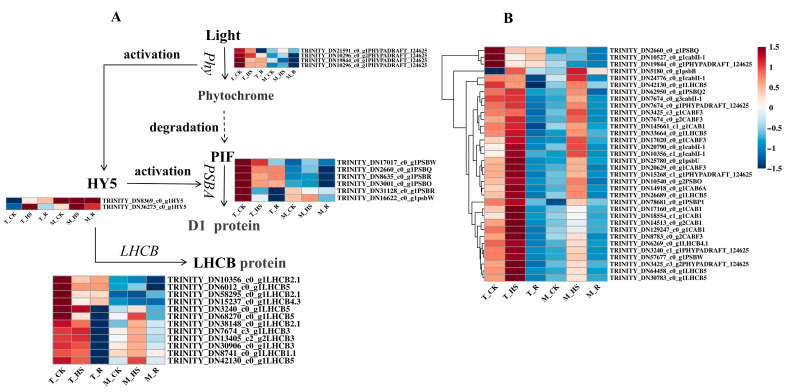
Heatmap of DEGs related to photosynthesis. (**A**) Pathways of photosynthesis. *Phy*, *phytochrome gene*; *PSBA*, *photosystem II protein D1 gene*; *LHCB*, *light-harvesting chlorophyll a/b-binding protein gene*; *HY5*, translation factor. (**B**) Heatmap of 34 key genes involved in the photosynthesis pathway.

A total of 221 DEGs involved in photosynthesis were identified from the genes involved in the photosynthesis of *E. nuttallii*. A total of 17 key genes regulating the photosynthesis process were screened out, including 6 *light-harvesting chlorophyll a/b-binding protein genes* (*LHCB*), 7 *photosystem II protein D1 genes* (*PSBA*), and 4 *phytochrome genes* (*Phy*) (Figure 6). *Phy*, *PSBA*, and *LHCB* genes were significantly downregulated in the T_HS. After removing temperature stress, most of these genes continued to be downregulated, except for one *Phy*, one *PSBA*, and two *LHCB* genes. In the M_HS, two *Phy*, three *PSBA*, and nine *LHCB* genes were upregulated, while *Phy*, *PSBA*, and *LHCB* genes showed continuous downregulation in the M_R.

### 3.8. Analysis of Differentially Expressed Genes Related to Sugar Metabolic Pathways

**Figure 7:**
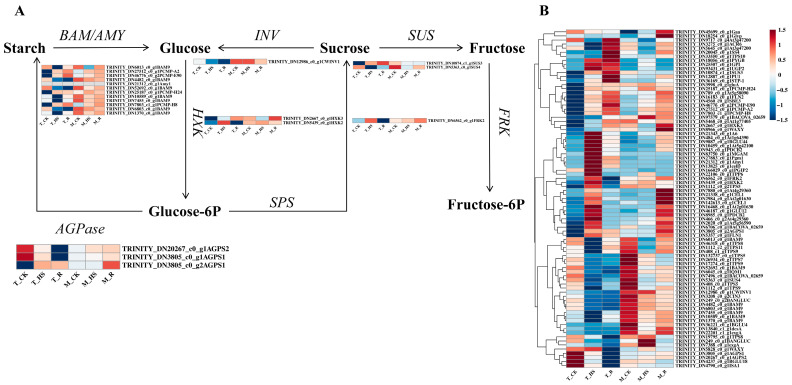
Heatmap of DEGs related to soluble sugar metabolic pathways. (**A**) Pathways of soluble sugar metabolism. *INV*, *invertase gene*; *SUS*, *sucrose synthase gene*; *HK*, *hexokinase gene*; *BAM/AMY*, *α-/β-amylases gene*; *FK*, *fructokinase gene*; *AGPase*, *ADP glucose pyrophosphorylase gene*; *SPS*, *sucrose phosphate synthetase gene*. (**B**) Heatmap of 83 key genes involved in the soluble sugar metabolic pathway.

SS are involved in osmotic regulation and sugar metabolism pathways, and their accumulation is mainly related to starch/sucrose metabolic pathways. We identified 83 DEGs involved in SS synthesis (Figure 7B), from which 32 key genes were screened. These genes included 3 *sucrose phosphate synthase genes* (*SUS*), 1 *fructokinase gene* (*FRK*), 2 *hexokinase genes* (*HXK*), 12 *α-/β-amylase genes* (*BAM/AMY*), 1 *invertase gene* (*INV*), and 2 *glucose pyrophosphorylase genes* (*AGPase*). Heatmap analysis revealed that in the T_HS, five *BAM/AMY*, one *SUS*, *FRK*, two *HXK*, and one *AGPase* were significantly upregulated, while three *BAM/AMY*, one *SUS*, and one *HXK* were downregulated among the upregulated genes in the T_R, and the other genes showed a continuous upward trend. In the T_HS, seven *BAM/AMY*, *INV*, one *SUS*, and one *AGPase* were significantly downregulated. In the T_R, three *BAM/AMY* and *INV* were upregulated, and the other genes showed a continuous downward trend. In the middle (M_HS), four *BAM/AMY*, one *HXK*, *FRK*, and three *AGPases* were significantly upregulated, and in the M_R, three *BAM/AMY*, *FRK*, and three *AGPases* were significantly downregulated. The other genes were continuously upregulated. In addition, seven *BAM/AMY*, one *HXK*, and two *SUS* were significantly downregulated, while two *BAM/AMY* and one *SUS* were upregulated, and the other genes were continuously downregulated (Figure 7A).

### 3.9. Analysis of Differentially Expressed Genes Related to Phenylpropanoid Biosynthesis Pathway

**Figure 8:**
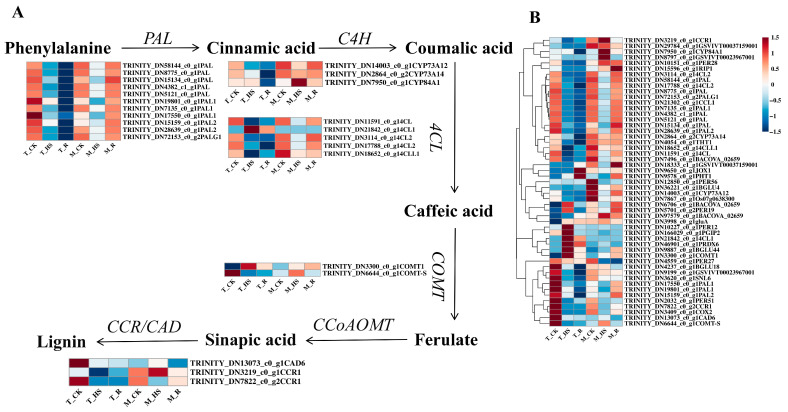
Heatmap of DEGs related to the phenylpropanoid biosynthesis pathway. (**A**) Pathway of phenylpropanoid biosynthesis. *PAL*, *phenylalanine ammonia-lyase gene*; *C4H*, *cinnamate-4-hydroxylase gene*; *4CL*, *4-coumarate: CoA ligase gene*; *COMT*, *caffeic acid O-methyltransferase gene*; *CCR*, *cinnamoyl CoA reductase gene*; *CAD*, *cinnamyl alcohol dehydrogenase gene*. (**B**) Heatmap of 51 key genes involved in the phenylpropanoid biosynthesis pathway.

The phenylpropanoid biosynthesis pathway synthesizes lignin and flavonoids to enhance plant defenses. Of the 51 DEGs identified (Figure 8B), 24 key genes were screened, including 11 *phenylalanine ammonia-lyase genes* (*PAL*), 3 *cinnamate-4-hydroxylase genes* (*C4H*), 5 *4-coumarate: CoA ligase genes* (4*CL*), 2 *caffeic acid O-methyltransferase genes* (*COMTs*), 2 *cinnamoyl CoA reductase genes* (*CCRs*), and 1 *cinnamyl alcohol dehydrogenase* (*CAD*). Except for one *C4H* gene, one 4*CL* gene, and one *COMT* gene in the T_HS, the other genes were significantly downregulated, and they continued to be downregulated in the T_R. Except for one *C4H* gene, 2 *COMT* genes, *CCR,* and one *CAD* gene in the M_HS, the rest showed a downward trend, and in the M_R, they showed an upward trend (Figure 8A).

### 3.10. Analysis of Differentially Expressed Genes Related to Heat-Shock Proteins (HSPs) and Heat-Shock Transcription Factors (HSFs)

**Figure 9:**
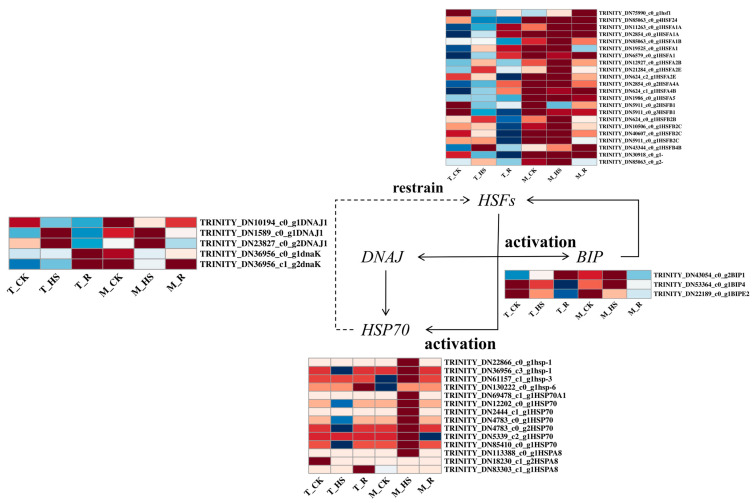
Heatmap of DEGs related to HSPs and HSFs. BIP, ER-resident HSP70 gene; DNAJ, HSP40 family gene; HSP70, HSP70 gene.

The biosynthesis of HSPs represents a critical regulatory response to thermal stress in plants. We identified 20 HSFs governing HSP regulation (Figure 9). Under HTS, multiple HSFs were upregulated in the T_HS, with significantly enhanced induction in the M_HS. Notably, the majority of HSFs exhibited constitutive regulation in the M_CK. Among the 28 differentially expressed HSP genes screened, 22 key candidates were prioritized: 3 BIP genes, 5 *DNAJ genes*, and 14 *HSP70 family members*. Thermopriming triggered significant upregulation of 2 *BIP genes*, 4 *DNAJ genes*, and 10 *HSP70 genes* in both the T_HS and M_HS groups, with consistently stronger induction magnitudes in the M_HS versus the T_HS.

### 3.11. Response of E. nuttallii to HTS

HTS induces the accumulation of reactive oxygen species (ROS) in *E. nuttallii*, triggering membrane lipid peroxidation. Structural alterations in plasma membrane proteins, including calcium channels, lead to changes in membrane fluidity. Calcium signaling plays a pivotal role in sensing temperature fluctuations and activating downstream signaling cascades. Transcription factors respond to thermal sensors by inducing HSP expression and targeting downstream regulators (e.g., *HsfA3*), thereby activating heat stress-responsive genes (including *Hsp70*, *sHsps*, and other molecular chaperones). These genes are essential for ROS scavenging and proteostasis maintenance. Additionally, transcription factor (TF) genes, such as *WRKY*, *bHLH*, and *MYB*, participate in the molecular response to thermal stress.

**Figure 10:**
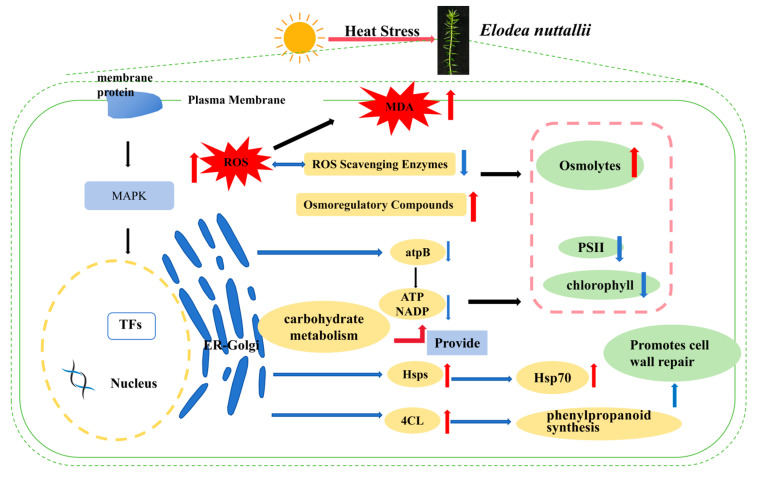
Response of *E. nuttallii* to HTS. The red up arrow indicates an increase and the blue down arrow indicates a decrease.

ROS accumulation elevates the activity of antioxidant enzymes (e.g., APX and SOD). Osmoprotectants, including Pro, SS, and SP, accumulate to enhance osmotic adjustment and maintain cellular ion homeostasis. Concurrently, downregulation of photosynthesis-related genes (e.g., *atpB* encoding ATP synthase β-subunit) under high temperature suppresses photosynthetic efficiency. Reduced synthesis of ATP and NADPH significantly impairs carbohydrate and starch biosynthesis pathways. To sustain energy metabolism, *E. nuttallii* enhances carbohydrate metabolic processes, while the phenylpropanoid biosynthesis pathway upregulates genes (e.g., *4CL*) to promote lignin synthesis, flavonoid biosynthesis, and lipid production (Figure 10).

## 4. Discussion

The physiological characteristics of plants are often related to their homeostasis mechanisms under stress [35]. The relative growth rate of *E. nuttallii* was negatively correlated with temperature under HTS. Additionally, under HTS, the leaves of *E. nuttallii* curled, and the stems were damaged. Physiological parameter analysis revealed an increase in antioxidant enzyme activities, with significantly higher levels in the apical tissues compared to the central tissues. Antioxidant enzyme activities were more pronounced at higher temperatures than at lower temperatures; however, a reduction in activity was observed at 30 °C and 35 °C. This pattern reflects *E. nuttallii*’s adaptation to temperature stress, a characteristic that has been documented in previous studies [28]. The results of physiological indices showed that the chlorophyll content of *E. nuttallii* decreased significantly under HTS, which was consistent with the observed decreases in chl a and chl b contents under HTS in *Paeonia ostii* [36]. The results of the chlorophyll fluorescence assay showed that the Fv/Fm value exhibited a decreasing trend under HTS (Figure 4). In their study on the effect of HTS (40 °C) on the photosynthetic characteristics of *Paeonia suffruticosa* leaves, Wen et al. mentioned that the maximum photochemical efficiency (Fv/Fm) of *P. sinensis* gradually decreased under HTS, indicating that high temperatures destroyed the photosynthetic capacity of *P. sinensis* leaves and damaged their photosynthetic organs [37]. The results of transcriptome analysis showed that *Phy* in the photosynthesis pathway was significantly downregulated under HTS, resulting in the downregulation of the photoharvesting protein LHCB and the PSBA protein (Figure 6), thereby inhibiting the progress of photosynthesis. In Gupta’s study, the photosystem PSII of *Hymenaea courbaril* was forced to change its structure, resulting in a decrease in photosynthetic efficiency under non-stomatal confinement [38,39].

The decrease in ATP and NADP produced by the inhibition of photosynthesis indirectly leads to a decrease in starch and sucrose synthesis, and the plant body increases the decomposition of starch and sucrose to maintain energy supply. In Flávia Lourenço da Silva’s study, *Prunus persica* was shown to rely on rapid sugar decomposition and fermentation to maintain energy supply and adapt to stress under flooding stress [40], which was consistent with the transcriptome enrichment analysis of the starch/sucrose metabolism pathways in this experiment. In the T_HS, *BAM/AMY* was significantly upregulated in the M_HS to promote the decomposition of starch into glucose; *SUS* and *FRK* were upregulated to promote the decomposition of sucrose into fructose; and *AGPase* downregulation inhibited starch synthesis to reduce energy loss. Therefore, under HTS, *E. nuttallii* may achieve short-term dormancy by increasing the decomposition of starch and sucrose and maintaining the body’s energy supply. Moreover, SS participate in osmotic adjustment. Under HTS, fundamental differences exist in osmoregulatory functions between terrestrial and aquatic plants (particularly submerged species). Terrestrial plants maintain leaf–soil water potential gradients primarily through stomatal closure to reduce transpiration, whereas aquatic macrophytes—lacking this energy-efficient stomatal regulation pathway—must continuously synthesize organic osmolytes to sustain water potential. Consequently, thermostressed *E. nuttallii* exhibits enhanced starch catabolism, elevating glucose, fructose, and other soluble sugars and thereby significantly increasing SS accumulation. Concurrently, substantial Pro accumulation enables osmotic equilibrium maintenance through osmoregulation to mitigate thermal damage. These findings align with Shang et al.’s research on the aquaporin gene family dynamics in thermostressed *Spirodela polyrhiza* [41].

The accumulation of ROS in plants under HTS leads to membrane lipid peroxidation. Antioxidant enzymes can scavenge ROS, and the phenylpropanoid biosynthesis pathway can be activated under HTS to produce various phenolic compounds to scavenge ROS [42]. Flavonoids can enhance the ability to scavenge H_2_O_2_ and toxic hydroxyl radicals in plant cells [43]. Ortega et al. [44] found that phenolic compounds, including phenols, flavonoids, and phenylpropanoid glycosides, were involved in the defense response of sunflower, demonstrating that the phenylpropanoid biosynthetic pathway plays a key role in the response to HTS. The results of transcriptome analysis showed that multiple rate-limiting enzyme genes involved in phenylpropanoid biosynthesis were significantly upregulated under HTS, including *CYP84A1*, *4CL1*, *and COMT1* in the T_HS, and *CYP84A1*, *COMT-S*, *CAD6*, and *CCR1* in the M_HS (Figure 8); the upregulation of these genes increased lignin synthesis. Lignin is one of the main components of plant cell walls and can enhance the strength and toughness of the cell wall, thereby providing support and protection [45]. Botanically, regarding morphological characteristics, the apical meristematic region of the shoot tip is enriched with cells in the division and elongation phases. The cell walls of these meristematic cells are primarily composed of pectin and cellulose. In contrast, the central tissues consist predominantly of differentiated cells, with mature cell walls composed mainly of lignin and cellulose. The apex exhibits high sensitivity to HTS, mounting rapid responses to this stress and demonstrating swift recovery upon its removal. Conversely, the central tissues respond more slowly to HTS and exhibit a reduced capacity for recovery post-HTS compared to the apical tissues. In Yun’s study, HTS decreased the level of precursors needed for lignin synthesis (ferulic acid, sinapic acid, cinnamic acid, and caffeic acid) but increased lignin content, leading to an increase in *Citrus unshiu* Marc. peel hardness [46], so lignin synthesis in *E. nuttallii* increased under HTS, helping the plant enter a dormant state and enhancing its likelihood of survival.

Shi et al.’s study showed that perennials usually undergo dormancy during the hot summer months to increase their chances of survival [47]. Species also undergo summer bud dormancy in semi-arid regions where summers are extremely hot and dry. Dormancy in summer is a way to increase plant survival by reducing survival-related activities and thereby reducing water loss while redistributing carbohydrates to meristematic tissues to keep the plant alive and support its regeneration [48,49].

In this study, *E. nuttallii*, as a submerged plant, did not rely on dehydration avoidance or transpiration reduction mechanisms to resist HTS. Our results showed that under prolonged 35 °C/40 °C treatment, the antioxidant enzyme activities, MDA levels, and osmoregulatory substances in *E. nuttallii* first increased and then decreased (Figure 3, Figure 4 and Figure 5). Notably, the MDA content gradually returned to normal levels during the recovery phase after a 72 h continuous treatment (Figure 4), indicating that short-term heat stress activates a progressive dormancy mechanism through reduced metabolic activity to endure sustained thermal challenges. Following the removal of temperature stress, *E. nuttallii* exhibited a decrease in antioxidant enzyme activity, an increase in chlorophyll content, and an elevation in Fv/Fm values. This indicates the potential for damage repair and restoration of physiological activities in *E. nuttallii* after the cessation of temperature stress. Our transcriptomic analyses further revealed that genes regulating various metabolic pathways, such as the antioxidant enzyme gene *PER12*, photosynthesis *PETF*, and the osmoregulation-related gene *ALDH2*, were significantly upregulated under HTS at 35 °C and then rapidly recovered to downregulation within a short time after recovering under 25 °C conditions. In contrast, genes related to the phenylpropanoid biosynthesis pathway (*CAD6*) and those related to the sucrose/starch synthesis pathways (*AGPS2* and *ISA1*) were significantly downregulated under HTS at 35 °C but quickly returned to the control level after recovering at 25 °C. These coordinated responses demonstrate that *E. nuttallii* employs a temperature-responsive dormancy strategy by means of rapid physiological priming, coupled with sustained metabolic curtailment, to enhance its extreme temperature tolerance.

## 5. Conclusions

In this study, we found that HTS induces excessive accumulation of ROS in *E. nuttallii*, causing oxidative damage to plant cells and altering the expression levels of both enzymatic and non-enzymatic antioxidants. These changes subsequently suppress photosynthetic carbon fixation along with starch and sucrose metabolism. To counteract these effects, *E. nuttallii* employs a coordinated adaptive strategy: elevating antioxidant enzyme activity to scavenge ROS, enhancing starch and sucrose catabolism to sustain energy supply, and promoting lignin biosynthesis to reinforce cell wall defense structures, thereby achieving physiological dormancy under thermal stress. Our findings provide novel theoretical insights for elucidating the high-temperature dormancy mechanism in *E. nuttallii*. Prior to this study, research on the response mechanisms of *E. nuttallii* to HTS was limited. The present investigation integrated physiological and transcriptomic analyses, providing a more in-depth exploration of the HTS response mechanisms in *E. nuttallii*. Furthermore, several key genes involved in the regulatory processes were identified. Consequently, it is proposed that future research could build upon these findings to validate the candidate key genes, thereby laying the groundwork for novel strategies in breeding thermotolerant cultivars.

## Figures and Tables

**Table 1:** Changes in the AGR of *E. nuttallii* under various temperatures.

Time	25 °C	30 °C	35 °C	40 °C
24 h	0.49 ± 0.13 a	0.35 ± 0.07 b	0.21 ± 0.08 b	−0.04 ± 0.07 c
48 h	0.54 ± 0.12 a	0.22 ± 0.07 b	0.17 ± 0.05 b	−0.17 ± 0.05 c
72 h	0.66 ± 0.12 a	0.22 ± 0.05 b	0.15 ± 0.04 b	−0.23 ± 0.04 c
Recovery 25 °C 72 h	0.49 ± 0.06 a	0.2 ± 0.04 b	0.11 ± 0.02 c	−0.15 ± 0.02 d

Note: Different lowercase letters indicate a significant difference at *p* < 0.05.

## Data Availability

The data in this project have been archived at NCBI under BioProject PRJNA1253947 with BioSamples: TCK1: SAMN48092801; TCK2: SAMN48092802; TCK3: SAMN48092803; THS1: SAMN48092804; THS2: SAMN48092805; THS3: SAMN48092806; TR1: SAMN48092807; TR2: SAMN48092808; TR3: SAMN48092809; MCK1: SAMN48092810; MCK2: SAMN48092811; MCK3: SAMN48092812; MHS1: SAMN48092813; MHS2: SAMN48092814; MHS3: SAMN48092815; MR1: SAMN48092816; MR2: SAMN48092817; and MR3: SAMN48092818. The reads have been deposited in the NCBI Sequence Read Archive as study SUB15275360 with the following accession numbers: TCK1: SRR33291196; TCK2: SRR33291195; TCK3: SRR33291186; THS1: SRR33291185; THS2: SRR33291184; THS3: SRR33291183; TR1: SRR33291182; TR2: SRR33291181; TR3: SRR33291180; MCK1: SRR33291179; MCK2: SRR33291194; MCK3: SRR33291193; MHS1: SRR33291192; MHS2: SRR33291191; MHS3: SRR33291190; MR1: SRR33291189; MR2: SRR33291188; and MR3: SRR33291187.

## Supplementary Materials

The following supporting information can be downloaded at https://www.mdpi.com/article/10.3390/biology14080993/s1, Table S1: clean_data_stat. Table S2: Transcripts from splicing. Table S3: A total of 109,287 unigenes were annotated in the KEGG, NR, Swiss-Prot, GO, Pfam, and eggNOG databases. Table S4: SNP variation. Table S5: Gene expressions.
